# Daily Duration of Compression Treatment in Chronic Venous Disease Patients: A Systematic Review

**DOI:** 10.3390/jpm13091316

**Published:** 2023-08-27

**Authors:** Sevara Mirakhmedova, Amirkhan Amirkhanov, Evgenii Seliverstov, Oksana Efremova, Igor Zolotukhin

**Affiliations:** Department of Surgery, Pirogov Russian National Research Medical University, Moscow 117997, Russia; kelly.gellespy@gmail.com (S.M.); uzdenuzdenov@gmail.com (A.A.); flebolog@rambler.ru (E.S.); lpl2@yandex.ru (O.E.)

**Keywords:** chronic venous disease, compression treatment, compression stockings, treatment duration, regimen

## Abstract

**Background:** There are no data on the daily regimen of compression therapy in patients with chronic venous disease. This systematic review aimed to establish the optimal daily duration of compression treatment. **Methods:** A systematic search of CENTRAL and MEDLINE was performed to identify RCTs, non-RCTs, reviews, systematic reviews, meta-analyses, and guidelines evaluating the use of compression regimens in the treatment of varicose veins. **Results:** Thirty-two RCTs, three non-RCTs, four observational studies, and two crossover trials reporting the duration and regimes of compression treatment fulfilled the inclusion criteria. The daily duration of compression was reported in patients after invasive treatment, for venous ulcer treatment, in patients with venous symptoms. The quality of the studies varied. We could not conduct a meta-analysis due to the heterogeneity of the research data and their quality. Twenty-three studies reported results of compression usage after invasive procedures. Eight studies reported daily duration regimens in patients with venous ulcers. Nine studies reported the impact of compression on venous symptoms and/or edema or limb volume change. One study was conducted to assess if compression improves QoL in venous patients. While there was a clear difference found in the daily duration depending on the clinical scenario, no data in support of exact regimens were found. **Conclusions:** There are no reliable data supporting exact daily regimens of compression treatment in various cohorts of CVD patients.

## 1. Introduction

Chronic venous disease (CVD) is a highly prevalent condition that affects more than 50% of the adult population [1,2,3,4]. CVD may significantly worsen a patient’s quality of life (QoL) and may lead to severe trophic disorders if left untreated [5,6]. Many studies confirm the efficacy of elastic compression either prescribed alone [7,8,9,10] or after invasive treatment, i.e., thermal and non-thermal ablation, phlebectomy, and sclerotherapy [11,12,13,14], which is in concordance with physicians’ opinions based on practice observations. Compression treatment, which is supported by guidelines, is widely prescribed for CVD patients [15,16,17].

On the other hand, patients’ adherence to compression treatment is often poor [18]. Wearing compression garments often leads to significant discomfort due to itching, sweating, skin dryness, and irritation [19]. Adverse events of compression make patients less compliant with treatment [18,20,21,22]. One possible way to improve adherence could be the adjustment of a daytime compression regimen, i.e., determining an optimal daytime duration effective at controlling CVD symptoms and signs. Nevertheless, although guidelines recommend compression as a first-line treatment for many patients, they do not detail the optimal day duration of compression [15,16,17,23].

This study aimed to investigate the optimal duration and regimens of compression treatment in CVD patients.

## 2. Methods

### 2.1. Search Strategy

For reporting the study results, the Preferred Reporting Items for Systematic Reviews and Meta-Analyses (PRISMA) guidelines were used [24]. Randomized clinical trials (RTCs), non-RCTs, reviews, systematic reviews, meta-analyses, and guidelines were searched using the Cochrane Central Register of Controlled Trials (CENTRAL), and PubMed (MEDLINE) databases to identify studies that evaluated the use of compression hosiery in the treatment of varicose veins. In addition, references in identified or related publications were reviewed to highlight any additional studies.

Search strategy used in CENTRAL:

#1 “varicose”

#2 varicose vein$

#3 #1 or #2

#4 “compression”

#5 stocking$

#6 hosiery

#7 bandage$

#8 #4 or #5 or #6 or #7

#9 “duration”

#10 “regimen”

#11 “compliance”

#12 #9 or #10 or #11

#13 thrombosis

#14 ((#3 and #7) and #12) not #13

Search strategy used in PubMed:

((“compression” OR “stocking” OR “stockings” OR “hosiery” OR “bandage”) AND (“varicose” OR “varicose vein” OR “varicose veins” OR “chronic venous disease”) AND (“duration” OR “regimen” OR “compliance”)) NOT “thrombosis”. Filters applied: Clinical Trial, Meta-Analysis, Randomized Controlled Trial, Review, Systematic Review.

### 2.2. Inclusion Criteria

Full-text articles reporting the duration and regimes of compression treatment published in English and not limited by year were included.

### 2.3. Exclusion Criteria

Studies were excluded if they reported compression treatment for asymptomatic varicose veins or in CVD patients with coexisting peripheral arterial disease.

### 2.4. Data Extraction

Datasets included first author, year of publication, study design, description of the cohort by CEAP classification, basic description of the studied population, treatment methods, class of compression, compression regimes, and assessment period, and the study results were extracted by two researchers (S.M. and A.A.). All disagreements were resolved by adjudication by a third reviewer (I.A.).

### 2.5. Risk of Bias

Two authors (S.M. and E.S.) independently assessed the quality of the included studies. All disagreements were resolved in consensus or with a third author (O.E.). A revised Cochrane risk-of-bias tool for randomized trials (RoB 2) “https://sites.google.com/site/riskofbiastool/welcome/rob-2-0-tool/current-version-of-rob-2 (accessed on 26 August 2023)”, the Risk Of Bias In Non-Randomized Studies-of Interventions (ROBINS-I) tool “https://sites.google.com/site/riskofbiastool/welcome/home/current-version-of-robins-i (accessed on 26 July 2023)”, and RoB 2 for crossover trials “https://sites.google.com/site/riskofbiastool/welcome/rob-2-0-tool/rob-2-for-crossover-trials (accessed on 23 July 2023)” were used to determine the methodological quality of the studies.

### 2.6. Statistical Analysis

It was impossible to draw any statistical analysis due to the variability of the compression products used, the different regimes used, and the different outcomes assessed.

## 3. Results

### 3.1. Literature Search

**Figure 1:**
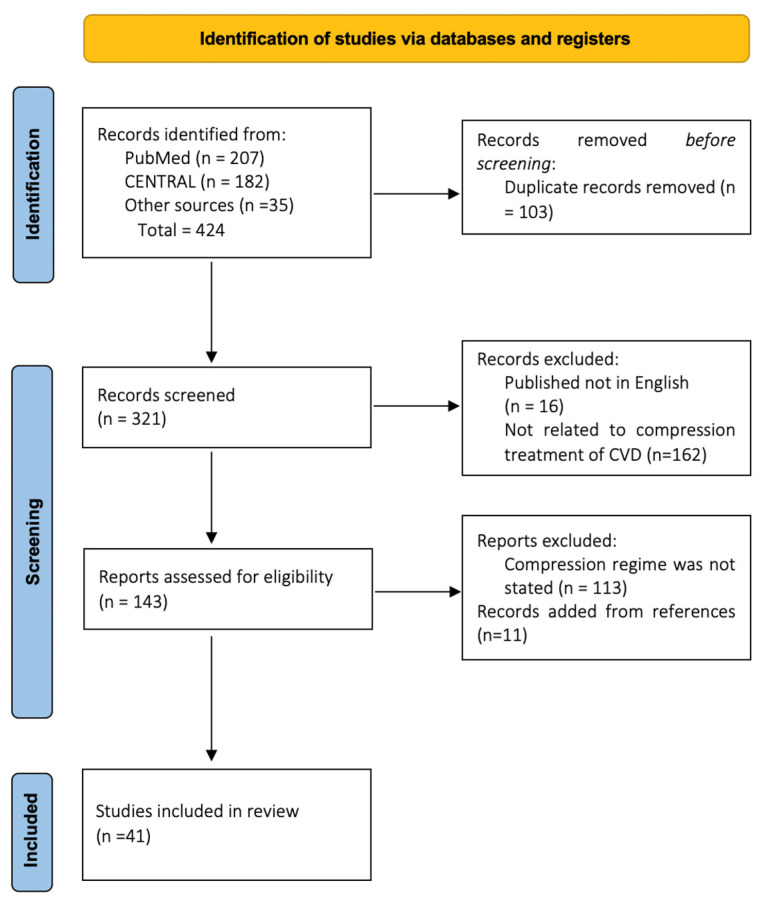
PRISMA flow chart of selection process.

A systematic review of the literature identified 424 potential records, of which 103 were rejected as duplicates, 16 as not reported in English, and 162 unacceptable after reviewing the title and abstract. Of the remaining 143 papers, a total of 41 papers were finally eligible for inclusion (Figure 1).

### 3.2. Included Studies

The analysis included 41 studies of which 32 were RCTs, three were non-RCT, four were observational studies, and two were crossover designed trials. The daily duration of compression was reported in patients after invasive treatment, for venous ulcers treatment, and in patients with venous symptoms. The main information extracted from the studies is presented in Table 1, Table 2, Table 3 and Table 4. 

### 3.3. Daily Duration of Compression after Invasive Procedures

Twenty-three studies reported the results of compression usage after invasive procedures including high ligation and stripping, phlebectomy, laser and radiofrequency ablation, and foam sclerotherapy (Table 1). For “daytime” and “during the day”, compression was prescribed in five [25,26,27,28,29] and two studies [30,31], respectively. The “day and night”, “24 h”, and “continuously” regimens were used in two [32,33], one [11], and two studies [34,35], respectively. In five studies, daily duration regimes changed consecutively, from “day and night” to “daytime” in two studies [36,37], from “day and night” to “8 h a day” in one [38], from “24 h” to “ daytime” in one [39], and from “24 h” to “while ambulatory” in one [40]. Other daily regimens used were “during walking hours” [41], “removed at night” [42], “throughout the day” [43], and “all day” [44]. In a study from Elderman et al., a mean daily duration of 12.48 h was registered [14]. Reich-Schupke et al. reported that, when patients were asked to use compression for the “whole day, for at least 8 h/day”, 29.3–42.6% of them wore stockings for 6–12 h a day, while 53.2–68% wore stockings for 12–18 h a day [45]. No comparison of the outcomes depending on different daily durations was made.

### 3.4. Daily Duration of Compression for Venous Ulcers’ Treatment

Eight studies reported daily duration regimens in patients with venous ulcers (Table 2). In two studies [46,47], the regimen was defined as “entire day”. Other regimes reported were “day and night” [48], “day and night plus walking hours” for 15 and 30 mmHg compression [49], “entire day, then removed at night till morning” [50], compression removed “at night” [51] or “at bedtime” [52], “24 h”, and “wakefulness” for different garments [53].

### 3.5. Daily Duration of Compression in Patients with Venous Symptoms/Edema

Nine studies were conducted to assess the impact of compression on venous symptoms and/or edema or limb volume change (Table 3). Four of them reported “8 h a day” as a prescribed regimen [54,55,56,57]. Other regimens used were “from morning to bedtime” [52,58], “during waking hours” [59], “from morning before going to bed” [60], and “day and night, then removed overnight” [61]. In the study of Belczak et al., there were two groups where Class-2 stockings were prescribed for 6 h/day and 10 h/day, while the third group did not use compression [62]. Evening edema was reduced in the treatment groups compared with no compression. Wearing stockings for 10 h/day was more effective than for 6 h/day.

One study was conducted to assess if compression improved QoL in venous patients. Patients were recommended to wear stockings no less than 8 h a day for six months. The authors measured the mean daily duration of compression treatment in patients with all C classes from C1 to C6. It varied from 9.33 to 10.4 h [63].

### 3.6. Risk of Bias

**Figure 2:**
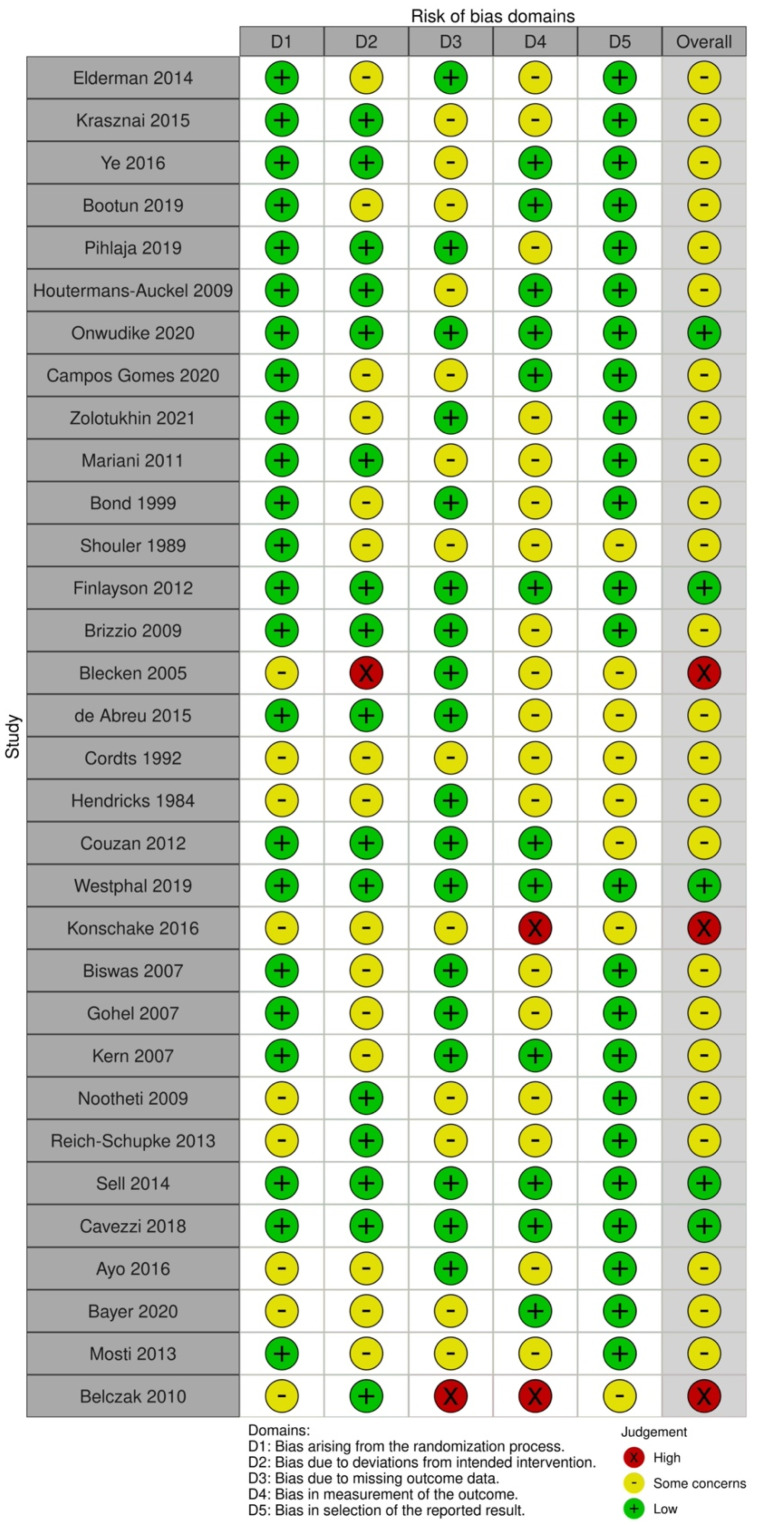
Quality assessment of included RCTs [11,14,25,26,27,30,31,32,33,34,35,36,37,38,39,40,41,42,43,44,45,46,47,48,50,51,52,55,56,58,61,62].

Twenty-four RCTs were labelled as having a moderate risk of bias (Figure 2) [11,14,25,26,27,30,31,32,33,34,35,36,38,40,41,42,43,45,47,48,50,52,58,61]. This was mainly due to no blinding of patients and/or researchers and a lack of descriptive information regarding study drop-out or withdrawals. Most studies were likely to include an appropriate randomization approach with attempts to create balanced groups at the baseline and to use allocation concealment and analysis by intention to treat. Five RCTs were classified as being at a low risk of bias overall, with all risk-of-bias domains were judged to be of low risk [37,39,44,51,55]. Three RCTs had a high overall risk of bias due to blinded outcome assessment [56,62], incomplete outcome data [62], and deviation from the treatment plan [46].

**Figure 3:**
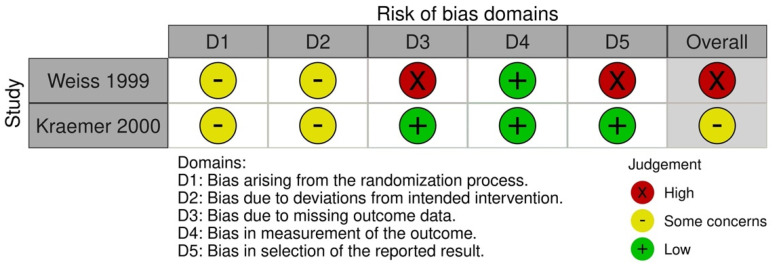
Quality assessment of included crossover trials [54,59].

Of the two crossover trials, one had a high risk of bias due to deviations from the intended interventions and the measurement of the outcome [59] (Figure 3). It was also unclear whether randomization was carried out. Another study had moderate risk and was designated as randomized, but the exact method was not specified [54].

**Figure 4:**
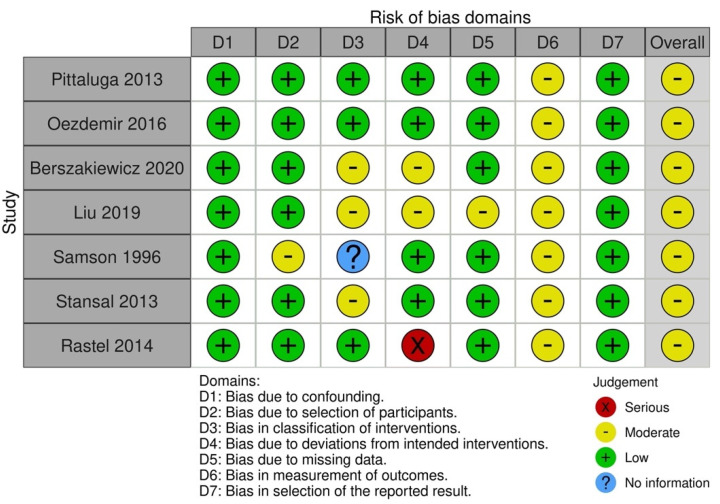
Quality assessment of non-RCTs [28,29,49,53,57,60,63].

All non-randomized studies had a moderate risk of bias (Figure 4). In all cases, there was a risk of bias due to the measurement of outcomes [28,29,49,53,57,60,63]. Four studies had risks associated with the classification of the interventions [29,49,53,63]. Three studies had domains alleged to be biased due to deviations from the intended interventions after assessment [29,57,63]. One study had bias regarding the selection of participants into the study [49], and the other one was due to missing data [29].

### 3.7. Meta-Analysis

Due to the heterogeneity of the data in terms of different indications for the compression treatment, regimes, and garments used and the differences in the outcomes reported, it was impossible to perform a meta-analysis to compare the results of different daily duration prescriptions.

## 4. Discussion

The present review was conducted to evaluate data on the impact of the daily duration of compression treatment in patients with CVD. To our knowledge, this is the first attempt to assess if treatment results depend on how many hours a day compression is prescribed.

This question arises from routine clinical practice observations and is supported by logical speculations. On the one hand, every patient for whom compression is prescribed asks a physician how long it has to be used during the day. No recommendations have been made by guidelines on this up to now. The decision is at the physicians’ discretion, whose recommendations are usually “for daytime”, “from wake up until going to bed”, etc. This prescription seems to be justified for patients with venous ulceration or severe edema. However, many patients experience signs and symptoms of much-less severity. For example, those who had deep venous thrombosis several months ago may have edema and symptoms, but no trophic disturbances. Compression may also be prescribed for symptomatic C1-C2 patients with a primary CVD. For such patients, the above-mentioned recommendations seem rather uncertain, confusing, and even redundant.

Daytime may significantly differ depending on geographical area and the season of the year. Waking time is an indefinite term also. Its duration may vary widely in patients of different ages, social statuses, occupations, physical conditions, etc. On the other hand, the lack of an exact daily “dosage” may lead to an increase in discomfort related to compression garments. Compression used from waking up until going to bed in the wintertime is not the same as in hot weather conditions. Practical observations confirm that patients’ compliance in warm weather is not good. Some patients report discomfort independently of environmental conditions, which makes them less compliant. What happens if compression is prescribed not for 15–16 h (from waking up till going to bed), but for 8–10 h a day, which is close to the average time of active orthostasis? It can be assumed that compression of a lesser duration would lead to less discomfort, thus increasing patients’ compliance with the treatment. To discuss if compression for several hours a day (and for how many hours) is reasonable, we should have data on whether this regimen is as effective as the commonly recommended daytime wearing. This systematic review was conducted to establish whether such data have been already obtained.

We found 40 studies in which daily duration of compression treatment were specified. Four groups of studies depending on the patients’ cohorts and end-points were identified. There were three main clinical scenarios, i.e., the period after invasive procedure, venous ulcer, or symptomatic CVD and/or edema. We also found one study that was designed to register QoL in all C classes.

**Figure 5:**
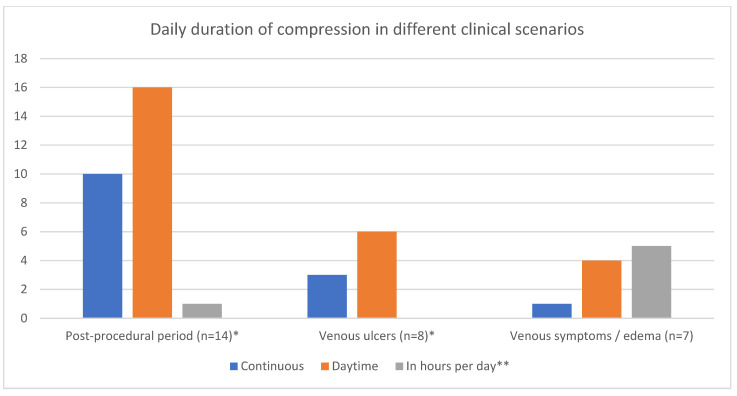
Daily duration of compression treatment in different clinical scenarios. * In some studies, more than one regimen was reported; ** with the exception of 24 h/day.

Interestingly, recommendations for the daily duration of compression were quite different (Figure 5). *Continuous (day and night, 24 h)* and *daytime (during the day, all day, etc.)* regimens were mainly used after invasive procedures and in venous ulcer patients. However, continuous compression was never prescribed for symptomatic patients and edema. A regimen with a duration *in hours per day* was used for symptomatic disease and edema control. In two other scenarios, such a regimen was used only once after an initial period of continuous compression. This reflects the difference in what the patients need in different clinical situations.

The post-procedural period is characterized by slight or moderate pain/discomfort, which may be reduced by elastic compression. Compression is also useful to control exudation from incisions in the first post-procedural days. This explains the prescription of compression for continuous use after invasive treatment. In a venous ulcer patient, the main goal of compression was to diminish the orthostatic loading of the venous system. Therefore, it is justifiable to recommend compression for the time when the patient is in the upright position, no matter whether standing or sitting, i.e., from waking up until going to bed. As for symptoms and/or edema control, it is generally assumed that it is enough to cover the working hours by compression. This leads to the use of daily dosage measured in hours, mainly 8 h a day, which is close to the common working day duration.

The general view of the findings from the literature confirms a connection between the daily duration of compression with the clinical situation. On the other hand, there are still no definite recommendations that can be used by physicians in routine clinical practice. The only regimen that is precisely defined with no interpretations possible is continuous (day and night, 24 h) compression.

When it comes to the daytime regimen, an important issue arises. Compression beyond nighttime was defined in fully different terms for patients who had undergone invasive treatment (daytime, during the day, whole day) and for those with ulceration (entire day, walking hours, compression removed at night at bedtime, wakefulness). This may indicate a need for different daily durations in these two cohorts. Interpreting daytime as working hours, i.e., from around 9 a.m. to around 5 p.m., needs exact definitions as patients may accept it their own way. For example, Elderman et al. prescribed daytime compression after EVLA and then measured the mean duration of wear. This resulted in 12.48 h a day [14]. This is also clearly different from what, for example, wakefulness means. In the study of Belczak et al., a duration of 10 h/day was also called “entire day” [62]. The nuances in using different terms cannot be caught by those physicians whose native language is not English. On the other hand, if one interprets daytime as the period between sunrise and sunset, then the geographical and seasonal conditions must also be taken into consideration. Thus, the lack of precise definitions of the mentioned terms may lead to a misinterpretation of the available data and the incorrect implementation in routine use of elastic compression.

In symptomatic CVD patients, the “dosage” in hours a day was most-often recommended. This seems quite reasonable, taking into account that venous symptoms are usually of light/moderate intensity and can be effectively managed by less-prolonged compression during the day. The goal of compression is to prevent symptoms from appearing by supporting venous return during orthostasis. The 8 h/day regimen reflects the common working day duration. However, no evidence exists about the effectiveness of this duration, especially in comparison with other regimens, i.e., 6, 10, or 12 h/day. The intensity of venous symptoms may differ, as well as the working day duration. It seems reasonable to adjust the daily duration accordingly. Belczak et al. found that a 10 h/day duration was more effective than 6 h/day in preventing edema after working hours in C0–C1 patients [62]. This is the only study that compared different regimens measured in hours a day. Thus, using compression 6 h/day seems to be insufficient in the early CVD stages. However, how many hours are enough for these patients? What is an optimal duration for C2–C3 patients—8, 10, 12, or more hours a day? Unfortunately, no studies have addressed this important practical issue yet.

Another issue related to the absence of definite recommendations is how patients follow physicians’ prescriptions. Being recommended to wear stockings no less than 8 h/day, C1 patients wore them 9.33 h/day on average, C2 9.3 h/day, C3 9.66 h/day, C4 10.2 h/day, C5 9.7 h/day, and C6 10.4 h/day [63]. In the study of Reich-Schupke et al., patients received the same recommendation to use compression for the “whole day, for at least 8 h/day”, but followed them differently. Some patients used compression for 6–12 h per day, while others wore stockings for 12–18 h per day [45]. It is clear that “daytime”, “entire day”, or “whole day” prescriptions are followed differently by patients.

### Limitations

There is only one study that reported the impact of different daily durations of compression treatment on the outcomes. The study had a high risk of biases. Furthermore, there are many definitions that are used to describe the same regimens. This makes it difficult to compare data from different studies. Moreover, patients’ compliance with the daily duration prescription was not measured objectively in either study. Improved reporting of daily compression regimens is essential to assess the treatment results.

## 5. Conclusions

This systematic review outlined the lack of data on the impact of different daily duration regimens on the effect of compression treatment in CVD patients. While, for different clinical scenarios, different duration regimens are used, no evidence exists of which regimen is optimal. The definitions of the same regimen may vary, which makes it difficult to compare the results from published studies. Future studies are needed to establish which exact daily duration of compression is optimal for patients with different clinical classes of CVD, especially for those with non-severe disease.

## Figures and Tables

**Table 1:** Compression after invasive procedures.

*Reference*	*Design*	*Patients*	*CEAP*	*Invasive Procedure*	*Study Groups*	*Compression Type*	*Daily Regimen*	*Overall Duration*	*Results*	*Intraday* *Duration (in Hours per Day If Specified)*	*Results Depending on Intraday Compression Regime*
*Bootun* et al. (*2019*) *[11]*	RCT	204	C2-5	RFA/EVLA	2 groups: compression vs. no compression after initial 24 h of wearing bandages	Class 2, thigh-length	Bandage for 24 h, then 24 h/day	7 days	Stockings are beneficial if phlebectomy is performed	24 h	NA
*Elderman* et al. (*2014*) *[14]*	RCT	79	C2-4	EVLA	2 groups: compression stockings vs. no compression after initial 24 h of wearing bandages	Class 2, thigh-length	Bandage for 24 h, then daytime (average: 12.48 h/day)	2 weeks	Post-procedural pain was slightly, but significantly less if stockings were used	Daytime, 12.48 h on average	NA
*Ye* et al. (*2016*) *[25]*	RCT	400	C2	EVLA	2 groups: compression vs. no compression	Class 2, thigh-length	Daytime (removed only at the measurement)	2 weeks	Stockings reduce pain and edema during the first week; no influence on QoL and mean time to return to work was found	Daytime	NA
*Pihlaja* et al. (*2019*) *[26]*	RCT	177	C2–4	RFA + FS	2 groups: compression vs. no compression	Class 2, thigh-length	Continuously for 2 days, then daytime for 5 days (removed only at the measurement)	7 days	No compression was non-inferior to compression in safety and efficacy	Daytime	NA
*Campos Gomes* et al. (*2020*) *[27]*	RCT	92 (135 legs)	C2–6	FS	2 groups: compression vs. no compression	Class 2, thigh-length	Bandage for 48 h, then daytime	3 weeks	Stockings in overweight patients did not decrease need for repeat procedures or the volume of foam	Daytime	NA
*Pittaluga* et al. (*2013*) *[28]*	nRCT	100	C2–6	Phlebectomy, ASVAL, stripping	2 groups: compression vs. compression (if it was necessary)	Class 2 (French), thigh-length	Daytime	8 days	No benefit from stockings beyond the first postoperative day for pain, ecchymosis, quality of life, and thrombosis	Daytime	NA
*Liu* et al. (*2019*) *[29]*	Retrospective Cohort Study	350 (377 legs)	C6	HL-EVLA-FS	2 groups: compression + surgery vs. compression alone	Class 2, thigh-length	Daytime	2 weeks	Combined treatment can shorten the ulcer healing time and reduce the ulcer recurrence rate compared with compression therapy alone	Daytime	NA
*Shouler* et al. (*1989*) *[30]*	RCT	99 (Group 1), 62 (Group 2)	NR	SF/SP ligation, phlebectomy	2 groups: VS + compression (2 subgroups: high vs. low) vs. sclero + compression (2 subgroups: stocking-only vs. stocking + bandage)	Low (15 mmHg)/high (40 mmHg) compression stockings, Elastocrepe bandage	During the day (Group 1), not to remove until interim review after 3 weeks (Group 2)	6 weeks	Low-compression stockings provided adequate support for after surgery and sclerotherapy; bandaging was not required if a high-compression stockings was used	During the day	NA
*Gohel* et al. (*2007*) *[31]*	RCT	500	C6	SFJ + GSV stripping to below the knee/SPJ disconnection, + varicosity avulsions	2 groups: compression vs. compression + VS	Open ulceration—multilayered compression bandaging (40 mmHg); healed legs—Class 2 stockings	During the day for Class-2 stockings	4 years	Surgical correction in addition to compression bandaging does not improve ulcer healing, but reduces the recurrence of ulcers at four years and results in a greater proportion of ulcer-free time	During the day	NA
*Krasznai* et al. (*2015*) *[32]*	RCT	101	C2-4	RFA	2 groups: compression 4 h vs. compression 72 h	Class 1 + Class 2, thigh-length	Postoperative 4 h vs. 72 h	NA	Stockings for 4 h after procedure are non-inferior in preventing leg edema as for 72 h	Day and night	NA
*Mariani* et al. (*2011*) *[33]*	RCT	60	C2	Stripping, perforating veins’ ligation	2 groups: compression stockings vs. short stretch compression bandages	Class 2, thigh-length (Sigvaris Postoperative Kit)	Day and night (only removed at the visits)	2 weeks	Short stretch bandage was better than stockings in terms of QoL, edema reduction, and compliance	Day and night	NA
*Bond* et al. (*1999*) *[34]*	RCT	42	NR	SFL + stripping, phlebectomy	3 groups: Panelast + TED vs. TED + MediPlus vs. MediPlus + Panelast	MediPlus Class 2 (30–40 mmHg), TED (10–12 mmHg), Panelast (30–38 mmHg)	Continuously	NR	No significant differences between the bandages were found	Continuously	NA
*Biswas* et al. (*2007*) *[35]*	RCT	220	C2–C4	SFJ flush-ligation + GSV PIN-stripping + phlebectomy	2 groups: compression for 1 week vs. compression for 3 weeks	Bandages, then standard full-length TED stockings	Continuously	1 week vs. 3 weeks	No benefit in wearing compression stockings for more than one week with respect to postoperative pain, number of complications, time to return to work, or patient satisfaction for up to 12 weeks following surgery	Continuously	NA
*Houtermans-Auckel* et al. (*2009*) *[36]*	RCT	104	C2-3	Crossectomy + short GSV stripping	2 groups: compression vs. no compression	Class 2, thigh-length	Bandage for 3 days, then day and night for 2 weeks, then daytime for 2 weeks	4 weeks	Compression had no additional benefit in limb edema, pain, complications, and return to work	Day and night, then daytime	NA
*Onwudike* et al. (*2020*) *[37]*	RCT	100	C2–5	RFA	2 groups: compression vs. no compression	Class 2, above knee	Day and night for 1 week, then daytime for 1 week	2 weeks	Compression had no additional benefit	Day and night, then daytime	NA
*Zolotukhin* et al. (*2021*) *[38]*	RCT	187	C2–4	RFA, phlebectomy	2 groups: compression stockings vs. compression sleeves	Class 2 compression sleeves vs. stockings for legs, thigh-length	Day and night for 7 days after the procedure, then >8 h daily	30 days	QoL improved equally in those who used compression sleeves and stockings	Day and night, then 8 h/day	NA
*Cavezzi* et al. (*2019*) *[39]*	RCT	94 (97 legs)	C2 and >	CFS + phlebectomy	2 groups: 23 mmHg compression (Group A) vs. 35 mmHg compression (Group B)	23 mmHg, thigh-length stockings vs. 35 mmHg, thigh-length, then thigh or panty stockings Class 1 (18–21 mmHg) for all patients	23/35 mmHg MCSs 24 h/day for 7 days, then Class 1 MCSs daytime for 33 days	7 days, then 33 days (40 days in total)	35 mmHg stockings provided less-adverse postoperative symptoms and better tissue healing; bioimpedance results confirmed a slightly better edema improvement with 35 mmHg medical compression stocking	24 h, then daytime	NA
*Nootheti* et al. (*2009*) *[40]*	RCT	29	C1	Sclerotherapy	2 groups: compression vs. no compression	Class 2 30–40 mmHg, thigh-high; Class 1 20–30 mmHg, thigh-high	24 h/day for 1 week Class 2, then 3 weeks Class 1 vs. 24 h/day for 1 week Class 2	4 weeks	Postsclerotherapy hyperpigmentation and bruising was significantly less with the addition of 3 weeks of Class I stockings	24 h, then while ambulatory	NA
*Ayo* et al. (*2016*) *[41]*	RCT	70 (85 legs)	C2-C5	EVLA and RFA	2 groups: compression vs. no compression after initial 24 h of wearing bandages	30–40 mmHg, thigh-high, bandages	Daily during waking hours	7 days	Compression therapy does not significantly affect both patients reported and clinical outcomes after GSV ablation in patients with non-ulcerated venous insufficiency	During waking hours	NA
*Kern* et al. (*2007*) *[42]*	RCT	96	C1	Sclerotherapy	2 groups: no compression vs. compression	23–32 mmHg, thigh-length stocking	Remove at night	3 weeks	Wearing compression stockings enhanced the efficacy of sclerotherapy of leg telangiectasias by improving clinical vessel disappearance	Remove at night	NA
*Bayer* et al. (*2020*) *[43]*	RCT	50 (100 legs)	C1	Sclerotherapy	2 groups: no compression after initial 24 h of wearing bandages (Group A) vs. compression (Group B)	Low-stretch bandages, 18–20 mmHg compression stockings, above the ankle	Continually every day throughout the day	1 week	One week of compression therapy had no clinical benefit compared with no compression	Throughout the day	NA
*Sell* et al. (*2014*) *[44]*	RCT	153	C2-C3	GSV ligation + PIN stripping/SSV ligation + PIN stripping/ AASV or PASV ligation, perforators, local phlebectomy	2 groups: superficial venous surgery vs. compression	Class 2 compression stockings	All day long during the upright position	2 years	Superficial venous surgery was better than compression stockings only	All day	NA
*Reich-Schupke* et al. (*2014*) *[45]*	RCT	88	NR	SFJ/SPJ flush-ligation + GSV/SSV stripping, phlebectomy	2 groups: low compression (Group A) vs. moderate compression (Group B)	18–21 mmHg/23–32 mmHg, thigh-high, compression stocking	Whole day, for at least 8 h/day (from the morning after getting up until going to bed)	6 weeks	23–32 mmHg compression contributes to a faster elimination of edema, pain, tightness, and discomfort in the early postoperative period, but no differences in the longer postoperative period were found	29.3% and 42.6% wore stockings for 6–12 h, 68% and 53.2% wore stockings for 12–18 h in Groups A and B, respectively	NA

AASV/PASV—anterior or posterior accessory saphenous vein; CFS—catheter foam sclerotherapy; EVLA—endovenous laser ablation; FS—foam sclerotherapy; GSV—great saphenous vein; HL-EVLA-FS—high ligation-endovenous laser ablation-foam sclerotherapy; RFA—radiofrequency ablation; SF/SP ligation—sapheno-femoral or sapheno-popliteal ligation; SFL—sapheno-femoral ligation; SFJ—sapheno-femoral junction; SPJ—sapheno-popliteal junction; SSV—small saphenous vein; VS—vein surgery.

**Table 2:** Compression in patients with venous ulcers.

*Reference*	*Design*	*Patients*	*CEAP*	*Study Groups*	*Ulcer Size*	*Ulcer Duration*	*Compression Type*	*Overall Duration*	*Results*	*Intraday* *Duration (in Hours per Day If Specified)*	*Results Depending on Intraday Compression Regimen*
*Blecken* et al. (*2005*) *[46]*	RCT	12 (24 legs)	C6s	2 groups: nonelastic compression (group A) vs. elastic compression (group B)	Group A: 48.98 ± 14.13 cm^2^ vs. Group B: 50.08 ± 18.30 cm^2^	NR	Nonelastic compression garment CircAid (group A) vs. Four-layer elastic bandage (group B)	12 weeks	Non-elastic garment is better than four-layer bandage in healing rate	Entire day	NA
*Cordts* et al. (*1992*) *[47]*	RCT	30	C6	2 groups: Unna’s boot vs. Duoderm CGF plus compression	9.1 cm^2^ for Duoderm group, 6.0 cm^2^ for Unna’s boot group (mean)	95 weeks (Duoderm group), 96 weeks (Unna’s boot group) (mean)	Unna’s boot vs. Duoderm + Coban wrap	12 weeks	Duoderm CGF HD is better than Unna’s boot	Entire day	NA
*Brizzio* et al. (*2009*) *[48]*	Single-center randomized open-label study	55	C6	2 groups: compression stockings vs. compression bandages	13 cm^2^ (mean)	27 months (mean)	MCSs 15–20 mm Hg + pads placed above incompetent perforating veins in the ulcer area vs. multi-layer short-stretch bandages + pads	13 weeks	Stockings and bandages are equally effective in pain relief.	Day and night	NA
*Samson* et al. (*1996*) *[49]*	Retrospective Cohort Study	53	C6	NR	300 mm^2^ (median)	UlcerCare system (Jobst, Toledo OH)	day and night 15 mmHg, walking hours of day 30 mmHg	6 weeks (median)	Continued stockings use after ulcer healing prevents most recurrences	Day and night, walking hours	NA
*de Abreu* et al. (*2015*) *[50]*	RCT	18	C6	2 groups: elastic bandage (group A) vs. Unna’s boot (group B)	15–28 cm^2^ (median)	NR	Elastic bandage (CCL 3) (group A) vs. Unna’s boot (group B)	13 weeks	The Unna Boot is better for >10cm^2^, ulcers, elastic bandage is better for <10 cm^2^ ulcers	Entire day, and remove at night till morning in group A	NA
*Finlayson* et al. (*2014*) *[51]*	RCT	103	C6	2 groups: compression vs. compression	4.1 cm^2^	23 weeks (median)	Four-layer bandage system vs. Class 3 hosiery	24 weeks	Stockings and bandages are equally effective	Compression was removed at night	NA
*Hendricks* et al. (*1985*) *[52]*	RCT *	21	C6	2 groups: compression vs. compression	NR	NR	Unna’s boot vs. open-toe, below the-knee, graded compression elastic support stockings (Futuro Style No. 50) (20–30 mmHg)	3–115 weeks	Healing rate is the same, while healing times is longer when stockings are used	Stockings were removed at bedtime	NA
*Stansal* et al. (*2013*) *[53]*	Prospective observational cohort study	89	C5–6	NR	NR	Class 1, 2, 3 or higher, single-layer bandage; multi-layer bandage	24 h—for short-stretch bandages, wakefulness—for long-stretch bandages and hosiery	NR	The healing rate was not assessed. Only half of the patients used compression correctly. Only a third complied with regime recommended	24 h (short-stretch bandages), wakefulness (long-stretch bandages)	NA

* Not specified in the published article; MCSs—medical compression stockings.

**Table 3:** Compression in symptomatic CVD and for edema or limb volume reduction.

*Author*	*Design*	*Patients*	*CEAP*	*Duration*	*Study Groups*	*Compression Type*	*Results*	*Intraday* *Duration (in Hours per Day If Specified)*	*Results Depending on Intraday Compression Regimen*
*Kraemer* et al. (*2000*) *[54]*	Crossover design—experimental prolonged standing	12 (24 legs)	C0S	28 days	3 groups: compression vs. compression vs. compression	Hosiery A, ankle: 7.7, calf: 7.6 thigh: 9.0 vs. Hosiery B, ankle: 7.6; calf: 6.8 thigh: 5.2 vs. Hosiery C, ankle: 15.4, calf: 8.4, thigh: 8.6 mmHg	Different compression stockings reduced leg discomfort and ankle edema	8 h	NA
*Westphal* et al. (*2019*) *[55]*	RCT	50	C1-4	28 days	2 groups: compression vs. skin care compression	Class 2, knee-high stocking (AD, Venotrain micro) (23–32 mmHg) vs. MCSs with integrated skincare substances (AD, Venotrain cocoon (23–32 mmHg)	Leg volume decreases under compression treatment	8 h	NA
*Konschake* et al. (*2016*) *[56]*	RCT	16	C3-6	1 week	3 groups: compression below-knee vs. compression thigh length vs. compression thigh-length	Below-knee two-component comp stockings (AD, Venotrain) (37 mmHg) vs. thigh-length two-component stockings (AG 37) (37 mmHg) vs. AG 45 (45 mmHg)	Thigh-length stockings were superior to below-knee stockings with regard to volume reduction, venous hemodynamics, and ulcer healing rates	8 h, inner liner of two-component stockings—during the daytime and night	NA
*Rastel* et al. (*2014*) *[57]*	Observational study	144	C2-C3	1 year	3 groups: daily wearing (>300 days/year; 8 h/day) vs. seasonal wearing (200–300 days/year; 8 h/day) vs. occasional wearing (<200 days/year; 8 h/day or 200–300 days/year; <8 h/day)	Eighty-nine patients bought and wore at least once the stockings: Class 1 (10–15 mmHg)—6 prescriptions, Class 2 (15–20 mmHg)—74 prescriptions, Class 3 (20–35 mmHg)—3 prescriptions, and unknown—6	Compliance with MCSs based on patients’ reported outcomes was low	8 h	NA
*Couzan* et al. (*2012*) *[58]*	Double-blind multicenter RCT	381	C2-5S	6 months	2 groups: progressive compressive stockings vs. degressive compressive stockings	Knee-length PCSs (10 mmHg at the ankle, 23 mmHg at the upper calf) vs. DCSs (30 mmHg at the ankle, 21 mmHg at the upper calf) (Lempy Medical, France)	Progressive stockings were more effective than degressive in pain control	From morning to bedtime	NA
*Weiss* et al. (*1999*) *[59]*	Prospective crossover trial	19 (38 legs)	C0-2S	4 weeks	1 group; no compression 2 weeks, compression 4 weeks	RTW lightweight gradient compression stockings (8–15 mmHg and 15–20 mmHg)	Low-compression hosiery improved venous symptoms	During waking hours	NA
*Oezdemir* et al. (*2016*) *[60]*	nRCT *	126	C2-3	4 weeks	2 groups: compression vs. no compression	Class 2, below-knee, 23–32 mmHg (JOBST brand)	Stockings improved disease-specific and general QoL by reducing venous symptoms	Wake up to before going to bed	NA
*Mosti* et al. (*2013*) *[61]*	RCT	28 (40 legs)	C3	4 weeks	2 groups: compression, inelastic bandage, then elastic stockings (Group A) vs. compression EK, then second stocking added (Group B)	Group A: inelastic bandage + short stretch nonadhesive bandage on the top, then 23–32 mmHg knee high-compression stockings; Group B: knee length grey “liner” from the Mediven Ulcer Kit, then second stocking of Mediven Plus Kit (20 mmHg) was added	The initial improvement in leg volume (1 week) was independent of the pressure applied, and the reduction was maintained by superimposing a second stocking	Day and night, then removed overnight	NA
*Belczak* et al. (*2010*) *[62]*	RCT *	20 (40 legs)	C0-1	NR	3 groups: compression entire day (10 h) vs. compression half a day (6 h) vs. no compression	Class 2, three-quarter-length, 20–30 mmHg	Stockings can be helpful in the reduction of evening edema during working hours	6 h (half day) 10 h (entire day)	10 h is better than 6 h

* Not specified in the published article; RTW—ready-to-wear; PCSs—progressive compressive stockings; DCSs—degressive compressive stockings.

**Table 4:** Compression treatment impact on QoL.

*Author*	*Design*	*Patients*	*CEAP*	*Duration*	*Study Groups*	*Compression Type*	*Results*	*Intraday* *Duration (in Hours per Day If Specified)*	*Results Depending on Intraday Compression Regime*
*Berszakiewicz* et al. (*2019*) *[63]*	Non-randomized comparative study	180	C1-6	6 months	6 groups: C1–C6	Prescribed ready-made compression hosiery (stockings, tights, knee-high socks)/prescribed ready-made 2-in-1 compression systems (VLUs)	Ready-made compression hosiery significantly improves the quality of life in C1–C6 patients	No less than 8 h/day C1—9.33 h C2—9.3 h C3—9.66 h C4—10.2 h C5—9.7 h C6—10.4 h	NA

## Data Availability

No new data were created or analyzed in this review. Data sharing is not applicable to this article.
